# Decrease of U(VI) Immobilization Capability of the Facultative Anaerobic Strain *Paenibacillus* sp. JG-TB8 under Anoxic Conditions Due to Strongly Reduced Phosphatase Activity

**DOI:** 10.1371/journal.pone.0102447

**Published:** 2014-08-26

**Authors:** Thomas Reitz, Andre Rossberg, Astrid Barkleit, Sonja Selenska-Pobell, Mohamed L. Merroun

**Affiliations:** 1 Helmholtz-Center Dresden-Rossendorf, Institute of Resource Ecology, Dresden, Germany; 2 Helmholtz-Center for Environmental Research, Department of Soil Ecology, Halle, Germany; 3 University of Granada, Department of Microbiology, Granada, Spain; Argonne National Laboratory, United States of America

## Abstract

Interactions of a facultative anaerobic bacterial isolate named *Paenibacillus* sp. JG-TB8 with U(VI) were studied under oxic and anoxic conditions in order to assess the influence of the oxygen-dependent cell metabolism on microbial uranium mobilization and immobilization. We demonstrated that aerobically and anaerobically grown cells of *Paenibacillus* sp. JG-TB8 accumulate uranium from aqueous solutions under acidic conditions (pH 2 to 6), under oxic and anoxic conditions. A combination of spectroscopic and microscopic methods revealed that the speciation of U(VI) associated with the cells of the strain depend on the pH as well as on the aeration conditions. At pH 2 and pH 3, uranium was exclusively bound by organic phosphate groups provided by cellular components, independently on the aeration conditions. At higher pH values, a part (pH 4.5) or the total amount (pH 6) of the dissolved uranium was precipitated under oxic conditions in a meta-autunite-like uranyl phosphate mineral phase without supplying an additional organic phosphate substrate. In contrast to that, under anoxic conditions no mineral formation was observed at pH 4.5 and pH 6, which was clearly assigned to decreased orthophosphate release by the cells. This in turn was caused by a suppression of the indigenous phosphatase activity of the strain. The results demonstrate that changes in the metabolism of facultative anaerobic microorganisms caused by the presence or absence of oxygen can decisively influence U(VI) biomineralization.

## Introduction

The rapid industrial growth during the last century has introduced different types of pollutants in the environment. One of the big challenges of the third millennium is the management of radioactive waste and the protection of humans and the environment from its chemical and radiological toxicity [1]. The remediation procedures of uranium-polluted sites and waste piles in most cases only include a covering of the surface to prevent from direct radiation as well as from the release of radon gas or radioactive dusts. However, many rehabilitated sites are not sealed at the bottom, allowing a release of uranium from these tailings into the ground water by erosion and leaching processes. The negative effects of uranium on the structure and functions of ecosystems strongly depend on its mobility and bioavailability, which are in turn determined by its speciation and physicochemical form [2]. In nature uranium primarily occurs in the oxidation states +4 (UO_2_) and +6 (UO_2_^2+^). In particular the uranyl ion (UO_2_^2+^), which predominates under acidic (<pH 5) and non-reducing conditions [3], has a high mobility and biological toxicity, which is based on both, its chemical and radiological properties. The speciation and therefore the mobility of uranium in nature is affected by a variety of abiotic [4]–[5] and biotic [6]–[7] factors. An important influence on the fate of uranium is exerted by microorganisms, which are present in large numbers and a great variety in nature. The multifaceted metabolism and structural components of these organisms provide various interaction mechanisms with radionuclides and other heavy metals [6], [8]. By now several studies have demonstrated that U(VI) is complexed by the negatively charged functional groups of the cell surface in a process called biosorption [9]–[11]. For this reason, uranium is supposed to bind, at least to some extent, to all microbial cells. In addition, further microbial U(VI) transformations which alter the mobility of this radionuclide were demonstrated for particular microbial genera and individual strains. One of these processes is the U(VI) bioreduction, i.e. the formation of low soluble and therefore less toxic U(IV) species [12]–[13]. However, the predominantly formed uraninite is not very stable in nature and can be easily re-oxidized. Another U(VI) immobilization process, which possesses a high potential for bioremediation of contaminated sites [14], is the U(VI) biomineralization. It includes the precipitation of insoluble mineral phases, formed due to the release of microbially-generated inorganic ligands [15]–[17]. A well-studied mechanism of uranium biomineralization is based on the activity of phosphatases [14], [18]–[19]. These microbial enzymes are expressed by a large variety of aerobic and anaerobic bacteria [20]–[22] and release inorganic orthophosphate from organic phosphate compounds. The released orthophosphate interacts with uranium and causes the precipitation of inorganic uranyl phosphate phases. As these precipitates are stable and poorly soluble in a wide range of pH, the formation of uranyl phosphate minerals can strongly decrease the mobility of uranium in nature.

Many of the before cited studies on U(VI)/microbe interactions revealed that underlying mechanisms strongly depend on abiotic parameters, such as pH, the ionic strength and the concentration of uranium. However, there is a lack of knowledge in the influence of another parameter, namely the oxygen tension, on U(VI)/microbe interactions. Even though it is well known that the presence or absence of oxygen has a strong influence on the cell metabolism and the uranium speciation itself, studies on the of U(VI)/microbe interactions in dependency on the oxygen tension are rare. The oxygen tension can, even quickly, change in soils and waters. In particular the upper soil layers continually experience cyclic changes in their oxygen tension due to changes in their moisture regime caused by rainfall events. Facultative anaerobic microorganisms are well adapted to such changes by switching their metabolism according to the presence or absence of oxygen. In this study we used the facultative anaerobic bacterium JG-TB8, which was recovered from a soil sample of the uranium mining waste pile “Haberland” (Johanngeorgenstadt, Saxony, Germany).

The main objective of the present study was to investigate the interactions of the facultative anaerobic isolate JG-TB8 with U(VI) under oxic and anoxic conditions, in order to determine the influence of the different cell metabolism on the uranium immobilization by this strain. Moreover, we studied the pH-dependency of the U(VI)/bacteria interactions under both aeration conditions. The aim was to determine the incidence of uranium biosorption, biomineralization, and bioreduction in dependency on the pH and the cell metabolism, which is in turn determined by the aeration conditions. The study contributes to a better understanding of the uranium mobility in contaminated ecosystems, which is needed to allow proper and reliable modelling for disposition of nuclear waste over many thousands of years as well as to develop new and to improve established bioremediation strategies for contaminated sites.

## Materials and Methods

### Isolation, phylogenetic affiliation and cultivation

*Paenibacillus* sp. JG-TB8 was isolated from an anaerobic microbial consortium enriched in liquid ATCC medium 591 [24] at pH 4.5. The microbial consortium was recovered from a soil sample of the uranium mining waste pile “Haberland” in Saxony, Germany having an acidic pH of 4.5 [25]. From this consortium facultative anaerobic strains were isolated by spreading the diluted consortium on Nutrient Broth Agar (Mast Group Ldt., Merseyside, UK). After incubation at 30°C for five days, single colonies were isolated, maintained in the same medium and long-term stored at −70°C in 50% glycerol. JG-TB8 was phylogenetically affiliated with *Paenibacillus* [23] on the basis of its 16S rRNA gene sequence obtained by using the primers pair 16S_7-27F_ and 16S_1492-1513R_. The 16S rRNA gene sequence of JG-TB8 has been submitted to the European Nucleotide Archive (http://www.ebi.ac.uk) and was assigned to the accession number FR849920. For biomass production the strain was grown aerobically in 10 g/L Nutrient Broth medium using a 5L bioreactor (Pilot System, Applikon Biotechnology Inc., Foster City, USA) equipped with a bio-controller (Model ADI 1010), a control unit (ADI 1075) with three pneumatic pumps (acid, base, and antifoam), a stirrer controller, and rota meters. Anaerobic growth of JG-TB8 was carried out in airtight closed two litre bottles in ATCC medium 591 [24]. In both cases, the cultivation temperature was 30°C and the pH was 7.2 as these parameters allowed rapid aerobic as well as anaerobic growth. Growth was monitored by measuring the optical density at a wavelength of 600 nm and cell morphology was examined by phase-contrast light microscopy using Olympus BX-61 (Olympus Optical Co. GmbH, Hamburg, Germany).

### Treatments with U(VI)

The anoxic treatments which are described in the following sections were performed in a glove box under nitrogen atmosphere (<10 ppm O_2_) and all required solutions were deoxygenated by five cycles of degassing under vacuum and subsequent flushing with nitrogen.

Cells in the logarithmic growth phase, i.e. at an OD_600_ of ∼0.6 for aerobic and ∼0.35 for anaerobic growth conditions, respectively, were harvested by centrifugation (10000 g, 15 min) and parallel portions were rinsed twice with 0.1 M NaClO_4_ at pH 2, 3, 4.5 or 6, respectively. Subsequently, the cells were suspended in 0.1M NaClO_4_; the pH of the cell suspensions was checked and if necessary adjusted to the desired values. U(VI) bioaccumulation studies were performed at room temperature using a biomass concentration of 0.5 g dry biomass (which was determined by weighing parallel samples after drying for 48 hours at 70°C) per litre. The microbial cells were incubated in triplicate on a rotary shaker with a 5×10^−4^ M uranyl nitrate, dissolved in 0.1 M NaClO_4_ (pH 2, 3 or 4.5). At pH 6 the uranium concentration was reduced to 5×10^−5^ M in order to prevent the formation of uranyl hydroxide precipitates. The uranium accumulation by the cells was determined in dependency on the incubation time and the pH of the solution. An incubation time of one hour was chosen for determining the U(VI) binding capacity bound by the cells exclusively *via* metabolism-independent biosorption. In order to assess further interaction mechanisms we additionally determined the U(VI) accumulation by the cells after incubation for 48 hours. For quantification of the uranium binding the bacterial cells were removed from the solution by centrifugation and the unbound U(VI) in the supernatant was measured by inductively-coupled-plasma mass-spectroscopy using an Elan 9000 system (Perkin Elmer, Waltham, MA, USA). The amount of removed uranium from the solution was normalized to the dry biomass.

### Time-Resolved Laser-induced Fluorescence Spectroscopy (TRLFS)

For TRLFS measurements portions (50 mg) of fresh grown cells were harvested, washed and treated with U(VI) for 48 hours at pH 2, 3, 4.5 or 6 under oxic conditions if grown aerobically, and under anoxic conditions if grown anaerobically, respectively. After the contact with U(VI) the cells were washed twice with 0.1 M NaClO_4_ solutions with the corresponding pH and under corresponding atmospheric conditions to remove unbound uranium. The samples were air-dried (anoxic samples under nitrogen atmosphere) for 48 h and subsequently ground. In order to determine the luminescence properties of the uranium complexes formed by the cells of JG-TB8, small portions of the dried and powdered samples were put into quartz micro cuvettes. Each of the anoxic samples was transferred immediately before the TRLFS measurement under nitrogen atmosphere into a quartz micro cuvette, which was subsequently closed airtight. Uranium luminescence was excited at 410 nm using a pulsed Nd-YAG laser (GCR 190, Spectra Physics, Santa Clara, CA, USA) [26]. The central wavelength of the spectrograph and the gate time of the ICCD camera were set to 520 nm and 50 µs, respectively. TRLF spectra were recorded between 444 and 594 nm with a resolution of 0.3 nm. Calibration of the spectrograph and background correction was performed (for details see [27]).

After U(VI) excitation, 101 U(VI) luminescence spectra (each calculated by averaging three single measurements) were recorded after defined delay times. For each sample two series of measurements differing in the delay times were performed. Delay time's step sizes were 200 ns and 1% of the time at which the complete U(VI) luminescence had faded away to determine uranium species exhibiting short and long luminescence lifetimes in the samples, respectively. The luminescence emission spectra were deconvoluted with the PeakFit module 4.0 of the software package Origin 8.0 (OriginLab Corporation, Northampton, MA, USA). Gaussian functions were used to describe the individual peaks and the parameters which were varied during the fitting procedure were peak wavelength, peak height and full width at half maximum. The luminescence lifetimes of the uranium complexes were calculated with exponential decay functions included in the software package.

### X-ray Absorption Spectroscopy (XAS)

Portions of the dried and powdered TRLFS samples were used for XAS measurements. The samples incubated under oxic conditions were mounted on Kapton tape as described earlier [26]. In the case of the samples incubated under anoxic conditions, the powdered biomass was transferred into specifically designed, heat sealed, polyethylene sample holders and subsequently stored in liquid nitrogen.

Two solutions, one of U(VI) and another one of U(IV), each at a concentration of 4×10^−2^ M in 1 M HClO_4_ served as reference samples for the uranium oxidation states. The stock solution of U(VI) was obtained by dissolving Na_2_U_2_O_7_×6 H_2_O in 7 M HClO_4_. Part of this solution was reduced electrochemically to U(IV) at a mercury pool cathode. The uranium oxidation state in these solutions was confirmed by UV/Vis spectroscopy.

The XAS measurements were performed at the ROssendorf BeamLine (ROBL) at the European Synchrotron Radiation Facility (ESRF), Grenoble, France [28]. Samples were measured at room temperature in fluorescence mode using a Si(111) double-crystal monochromator and a 13-element germanium fluorescence detector and analyzed with the software EXAFSPAK according to Reitz et al. [27]. The theoretical scattering phase and amplitude functions were calculated from structural models via the software FEFF8.2 [29]. All fits included the four-legged multiple scattering (MS) path of the uranyl group, U-O_ax_-U-O_ax_. The coordination number (N) of this MS path was linked to the N of the single-scattering (SS) path U-O_ax_. The radial distance (R) and Debye-Waller factor (σ^2^) of the MS path were linked at twice the R and σ^2^ of the SS path U-O_ax_, respectively [30]. During the fitting procedure, N of the U-O_ax_ SS path was held constant at two. The amplitude reduction factor was held constant at 1.0 for FEFF8.2 calculations and EXAFS fits. The shift in threshold energy, ΔE_0_, was varied as a global parameter in the fits.

### Transmission electron microscopy

The cellular localization of the uranium complexes formed by the cells of *Paenibacillus* sp. JG-TB8 was performed by using Transmission Electron Microscopy (TEM) combined with Energy Dispersive X-ray spectroscopy (EDX) for elemental analyses. After the contact with U(VI) for 48 hours the cells were rinsed twice with 0.1 M NaClO_4_ (pH 2, 3, 4.5 or 6). A third washing step was performed with 0.1 M sodium cacodylate buffer (pH 7.2). TEM samples were prepared according to former protocols [27] and analyzed using a “High Resolution Philips CM 200” transmission electron microscope at the “Centro de Instrumentatión Científica” of the University of Granada (Spain) at an acceleration voltage of 200 kV. EDX analyses were performed at the same voltage using a spot size of 70 Å and a live counting time of 200 s.

### Live Dead Staining

After the uranium treatments under the different experimental conditions for 48 hours, the cell suspensions were centrifuged at 4°C and 10000 g for 10 min. After that, the cells were washed twice with, and subsequently suspended in 330 µl 0.9% NaCl. 1 µl of the staining solution, containing a mixture of two fluorescence dyes (SYTO 9 and propidium iodide) (Live/Dead BacLightTM Bacterial Viability Kit L-7012, Molecular Probes, Inc., Eugene, OR, USA) was added to the samples. After incubation in the dark and on ice for 15 minutes unbound residues of the dye were removed by centrifugation. Cells were suspended in 25 µl 0.9% NaCl and samples were analysed using an Olympus BX-61 (Olympus Europa Holding GmbH, Hamburg, Germany) along with the imaging software “cell∧P”. Fluorescence was excited by light with wavelengths between 420 and 460 nm using a super-wide band filter mirror unit. The proportion of living cells in each sample was estimated by counting a total of 500 cells.

### Enzymatic assay

The phosphatase activity was determined by the “Acid Phosphatase Assay Kit” from Sigma-Aldrich (Saint Louis, MO, USA). Deviating from the manufacturer's instructions, *p*-nitrophenyl phosphate was not dissolved in water but in 0.1 M NaClO_4_ with pH 2, 3, 4.5 or 6, respectively, according to the conditions used for the U(VI) accumulation studies. For each pH value three samples, each containing 2 mg of freshly grown cells of JG-TB8 were washed four times with 0.1 M NaClO_4_ (pH 2, 3, 4.5 or 6). At each washing step, pH was controlled and if necessary readjusted to the required value. After that, the cells were suspended in 100 µl 0.1 M NaClO_4_ with the corresponding pH. The cells of three more samples were killed by heating at 121°C for 20 min and afterwards studied analogically at pH 6. 50 µl from each of the cell suspensions was transferred to 50 µl *p*-nitrophenyl phosphate solution with the same pH. Control reactions without cells were prepared to quantify the spontaneous hydrolysis of *p*-nitrophenyl phosphate. After incubation for 30 min at room temperature, the reaction was stopped by the addition of 200 µl 0.5 M NaOH. Subsequently, the cells were spun down and the supernatants of the samples were used to quantify the produced *p*-nitrophenol at 405 nm. Phosphatase activity in the different samples was calculated according to the protocol of the manufacturer.

### Colorimetric determination of phosphate

Phosphate was quantified by colorimetric measurements using malachite green. This dye complexes inorganic phosphate groups in the presence of molybdate and forms a complex which can be determined at a wavelength of 660 nm [31]. The phosphate reagent was prepared as described by Ekman and Jäger [32]. KH_2_PO_4_ solutions with concentrations ranging from 0 to 20 µM served as a standard. For the test samples each of about 5 mg of freshly grown cells were suspended in triplicate in 10 ml uranyl nitrate solutions. In addition, parallel samples without uranium were prepared. Samples were shaken for 48 hours at room temperature. After that, the samples were centrifuged and the amount of inorganic phosphate in the supernatant was determined. For this purpose 100 µl of the phosphate reagent were added to 100 µl of the cell supernatants as well as to the standard solutions. Samples were incubated for 20 min at room temperature and subsequently the absorption of the complex was measured at 660 nm and quantified *via* the determined standard curve.

## Results

### Phylogenetic affiliation and morphological characterization of JG-TB8

The 16S rRNA gene of the studied in this work bacterial isolate JG-TB8 shared 99.5% sequence identity with that of the bacterial strain *Paenibacillus* sp. 436-1, recovered from a soil sample from Wisconsin (USA) (Fig. S1 in File S1). In addition, JG-TB8 is closely related (97.5% sequence identity) to *Paenibacillus borealis*, strain DSM 13188^T^ ( = KK19^T^), a nitrogen-fixing strain isolated from an acid humus, collected in Finland [33].

Under aerobic growth conditions the cells of JG-TB8 were motile and rod-shaped, having a length of 3 to 6 µm and a width of 0.7 to 1 µm. (Fig. S2 in File S1, A1). In the stationary growth phase JG-TB8 produced ellipsoidal to rectangular endospores with a size of about 1×2 µm. The terminal swelled sporangia caused the club-shaped form which is typical for paenibacilli (Fig. S2 in File S1, A2). In contrast to that, under anaerobic growth conditions, no endospores were formed by JG-TB8 and connections of multiple cells to filamentous strands were observed (Fig. S2 in File S1, B1 and B2).

### Bioaccumulation of U(VI) by JG-TB8

As evident from the data presented in Table 1 the U(VI) accumulation by the strain *Paenibacillus* JG-TB8 under oxic as well as under anoxic conditions was pH-dependent and increased with increasing pH values (up to pH 4.5). The binding capacities obtained at pH 6 were not suitable for comparison, as the U(VI) accumulation was limited to about 24 mg uranium per g of dry biomass, due to the ten times lower U(VI) concentration in this sample. Apart from the samples incubated at pH 6, the U(VI) accumulation of the cells grown anaerobically and incubated under anoxic conditions was 25 to 35% lower compared to the U(VI) accumulation of cells grown aerobically and incubated under oxic conditions. After incubation for 48 h the U(VI) accumulation by the strain did not increase significantly at pH 2 and 3 under oxic conditions as well as under anoxic conditions. In contrast to that, at pH 4.5 under oxic conditions the amount of U(VI) associated with the cells approximately doubled. During the incubation the pH of the cell suspensions was checked. It remained stable (±0.2), besides at pH 6 (oxic and anoxic conditions) where the pH slightly increased to 6.3–6.5 within 48 h.

**Table 1:** U(VI) binding capacity calculated for the cells of JG-TB8 in dependency of pH conditions, presence of oxygen and incubation time.

Experimental conditions	U(VI) binding capacity [mg/g_dry_ _biomass_]^a^	
	1 hour	48 hours
JG-TB8 – pH 2.0 – oxic conditions	49.1±3.6	63.2±9.8
JG-TB8 – pH 3.0 – oxic conditions	58.7±4.7	69.1±8.8
JG-TB8 – pH 4.5 – oxic conditions	77.1±4.2	138±13
JG-TB8 – pH 6.0 – oxic conditions	24.6±1.3^b^	24.0±1.4^b^
JG-TB8 – pH 2.0 – anoxic conditions	31.5±6.6	34.8±8.5
JG-TB8 – pH 3.0 – anoxic conditions	41.5±4.7	47.3±7.3
JG-TB8 – pH 4.5 – anoxic conditions	58.6±9.0	66.4±3.4
JG-TB8 – pH 6.0 – anoxic conditions	23.8±1.1^b^	24.4±0.8^b^

a Experiments were performed in triplicate. The mean and the standard deviation are presented.

b At pH 6, the binding capacity is limited to ∼24 mg/g dry biomass due to the lower uranium concentration used in these samples.

### Speciation and molecular structure of the uranium complexes

#### TRLFS studies

**Figure 1:**
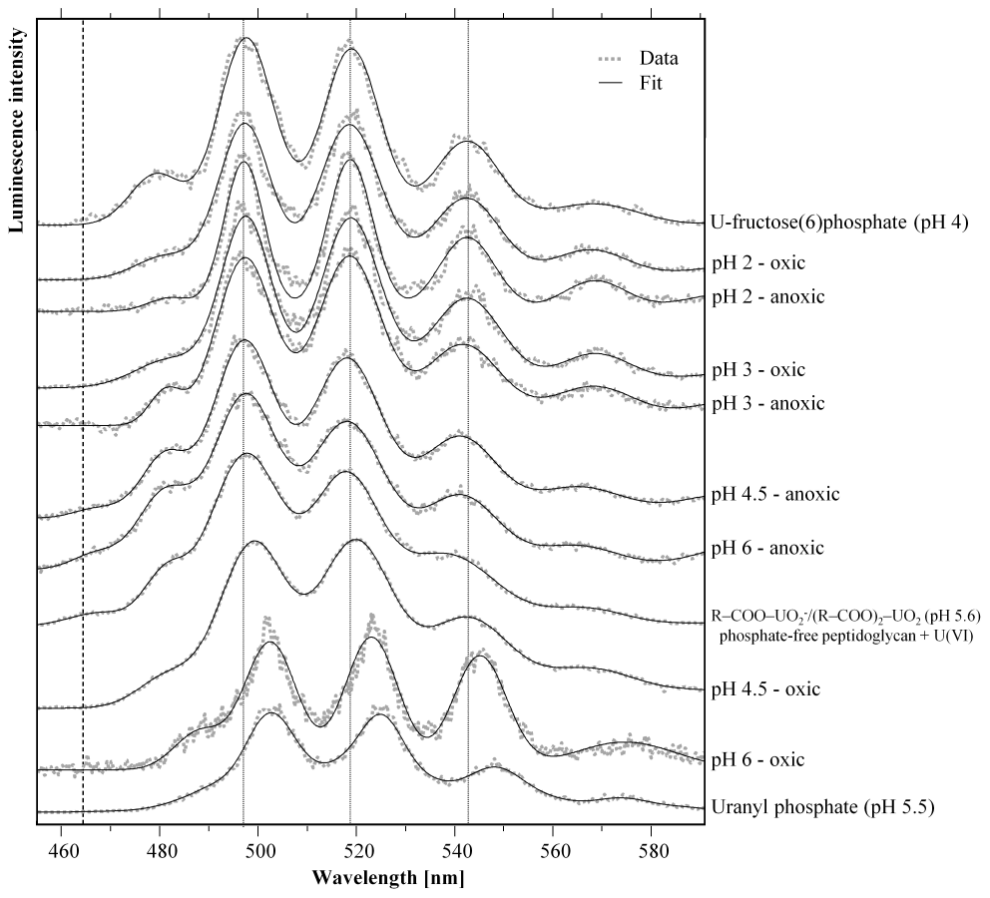
Normalized U(VI) luminescence spectra recorded from the uranium complexes formed under different pH and aeration conditions within 48 hours by the cells of *Paenibacillus* sp. JG-TB8. For comparison, dotted vertical lines indicate the main luminescence emission maxima recorded for the sample incubated at pH 2. The dashed vertical line marks the position of the luminescence emission peak which was assigned to uranyl carboxylate complexes.

In order to investigate the speciation of U(VI) associated with the cells, time-resolved laser-induced fluorescence spectroscopy (TRLFS) was performed. The recorded spectra are presented in Figure 1 and the corresponding peak maxima obtained by peak fitting procedures are summarized in Table 2. It is evident from the data that the U(VI) luminescence spectra at pH 2 and pH 3 in the oxic and anoxic samples do not differ from each other and consistently exhibit main emission maxima at around 497.3, 518.8, and 542.3 nm. These peak maxima correspond well to those of U(VI) complexes formed at phosphate groups of organic molecules, e.g. uranyl-fructose(6)phosphate (Fig. 1) and uranyl-adenosine monophosphate (Table 2). In addition, very similar TRLFS data were obtained in studies investigating the U(VI) complexation by isolated bacterial cell wall compounds (Table 2), such as glycerol-1-phosphate [36] as well as *o*-phosphoethanolamine (NH_3_CH_2_CH_2_OPO_3_^−^) and 1,2-dimyristoyl-sn-glycero-3-phosphate (DMGP) [37] that represent the polar head and the non-polar tail of phospholipids, the major components of cell membranes. Time-resolved analyses revealed a bi-exponential luminescence decay (Table 3) with luminescence lifetimes of around 3 µs (τ_1_) and 30 µs (τ_2_) in all samples incubated at pH 2 and pH 3. Due to the obvious formation of two different uranyl complexes we performed peak fits of luminescence spectra recorded after different delay times from each of the samples. However, we did not observe any shifts of the emission maxima, indicating that at pH 2 and pH 3 two different uranyl phosphate complexes with a high structural similarity were formed, independently of the aeration conditions.

**Table 2:** U(VI) luminescence emission maxima of the uranium complexes formed by *Paenibacillus* sp. JG-TB8.

Sample	Luminescence emission maxima^a^						Lifetime(s) (µs)
UO_2_^2+^ (pH 2)		473.0	**489.4**	**510.8**	**534.3**	559.7	1.98±0.11
***Paenibacillus*** ** JG-TB8 – aerobically grown/incubation with U(VI) under oxic conditions**							
U(VI)+JG-TB8 - pH 2.0		480.8	**497.2**	**518.7**	**542.3**	568.0	
U(VI)+.JG-TB8 - pH 3.0		481.1	**497.7**	**519.0**	**542.5**	569.0	Table 3
U(VI)+JG-TB8 - pH 4.5		481.7	**499.1**	**519.9**	**542.6**	567.8	
U(VI)+JG-TB8 - pH 6.0		488.1	**502.5**	**523.1**	**545.2**	574.0	
***Paenibacillus*** ** JG-TB8 – anaerobically grown/incubation with U(VI) under anoxic conditions**							
U(VI)+JG-TB8 - pH 2.0		482.3	**497.2**	**518.8**	**542.6**	568.6	
U(VI)+JG-TB8 - pH 3.0		481.3	**497.3**	**518.6**	**541.6**	568.3	Table 3
U(VI)+JG-TB8 - pH 4.5	467.5	480.9	**497.1**	**518.1**	**541.1**	565.3	
U(VI)+JG-TB8 - pH 6.0	468.2	481.0	**497.2**	**518.3**	**541.2**	564.9	
**Uranyl phosphate complexes**							
UO_2_-fructose(6)phosphate [34]		478.9	**497.1**	**519.0**	**543.3**	568.9	0.13±0.05
UO_2_-AMP [35]			**497**	**519**	**542**	569	n.d.
UO_2_-glycerol-1-phosphate [36]			**497.2**	**519.0**	**543.3**	568.9	0.15±0.03
UO_2_-DMGP [37]		481.5	**497.4**	**519.3**	**542.4**	567.5	1.0; 20
UO_2_-[NH_3_CH_2_CH_2_OPO_3_]^+^ [37]		483	**498.0**	**518.4**	**541.3**	565.9	3.1±0.6
R-O-PO_3_-UO_2_ [38]		481.5	**498.1**	**519.6**	**542.9**	567.5	1.2±0.4
**Uranyl phosphate minerals**							
Saleeite [41]		489.0	**501.1**	**522.1**	**545.7**	570.9	2.3±0.2
meta-autunite [41]		491.3	**501.8**	**522.9**	**546.9**	572.2	0.74±0.1
**Uranyl carboxylate complexes**							
Uranyl acetate [40]	462.9		**494.6**	**514.3**	**535.9**		
R–COO–UO_2_^+^/(R-COO)_2_-UO_2_ [39]	466.0	481.6	**498.1**	**518.0**	**539.0**	566.0	7.3; 0.7

a Main luminescence emission bands were pointed out by bold letters.

For comparison the U(VI) luminescence emission maxima of some model compounds, as well as those of the complexes formed by other microbial strains are presented for comparison.

**Table 3:** Calculated luminescence lifetimes of the U(VI) complexes formed by the cells of *Paenibacillus* sp. JG-TB8.

Lifetime	τ_1_	τ_2_	τ_3_	τ_4_	τ_5_	τ_6_
**pH 2.0 – oxic**	2.71±0.58 µs	26.7±4.4 µs				
**pH 3.0 – oxic**	2.59±0.25 µs	26.3±3.8 µs				
**pH 4.5 – oxic**		29.6±3.0 µs	120±5 µs	2.58±0.50 µs		
**pH 6.0 – oxic**					3.05±0.24 µs	
**pH 2.0 – anoxic**	3.32±0.87 µs	33.4±4.0 µs				
**pH 3.0 – anoxic**	3.62±0.45 µs	32.7±3.2 µs				
**pH 4.5 – anoxic**	2.70±0.39 µs	36.6±4.5 µs				7.82±1.25 µs
**pH 6.0 - anoxic**	2.99±0.24 µs	40.4±3.8 µs				9.21±1.09 µs

τ_1_, τ_2_, τ_3_: organic uranyl phosphate complexes.

τ_4_: mixture of organic and inorganic uranyl phosphate complexes.

τ_5_: inorganic uranyl phosphate complexes.

τ_6_: organic uranyl carboxylate complexes.

The luminescence spectra of the U(VI) associated with the cells at pH of 4.5 and pH 6 under oxic and anoxic conditions differed from those of the highly acidic samples incubated at pH 2 and pH 3. In the spectra of the anoxic sample (pH 4.5 and 6) an additional luminescence peak at around 468 nm occurred (Fig. 1) and, moreover, an additional U(VI) luminescence lifetime was calculated (Table 3). This weak luminescence peak was related to U(VI) complexes formed at carboxylic groups of organic compounds (compare the reference samples in Fig. 1 and Table 2). The observed luminescence lifetime of these uranyl carboxylate complexes (τ_6_ = 7.82±1.25 µs) is similar to those of the uranyl carboxylate complexes recently published by Barkleit *et al.* [39] and Vogel *et al.* [40].

In contrast to that, no corresponding peak was observed in the spectrum of the oxic samples (pH 4.5 and pH 6). Instead, the luminescence spectra were shifted to higher wavelengths (Fig. 1). At pH 6 the main emission bands were located at 502.5, 523.1, and 545.2 nm. In this sample the mono-exponential luminescence decay indicated the presence of only one predominant luminescent U(VI) species with a lifetime of τ_5_ = 3.05±0.24 µs (Table 3). Similar luminescence properties have been described for different natural and synthesized uranyl phosphate minerals such as saleeite or meta-autunite [41]. At pH 4.5 the emission peaks were poorly resolved and only slightly red-shifted in comparison to the acidic samples. This was most likely caused by the formation of inorganic and organic uranyl phosphate complexes under these conditions. However, due to the similar luminescence lifetimes of the U(VI) complexes formed with organic and inorganic phosphates (compare τ_1_ and τ_5_ in Table 3) the emission bands of these two species could not be separated from each other. The presence of the third, very long-living U(VI) species (τ_3_) was obviously not responsible for the observed differences in the shape of the spectrum as analyses of spectra recorded after longer delay times demonstrated emission bands highly comparable to those of the complexes formed at pH 2 and pH 3.

Whereas under anoxic conditions the luminescence properties of the sample incubated at pH 6 were very similar to those of the sample incubated at pH 4.5 (Fig. 1, Table 2), the U(VI) luminescence spectrum recorded from the sample incubated at pH 6 under oxic conditions was further shifted to higher wavelengths (Fig. 1).

#### XAS analyses

**Figure 2:**
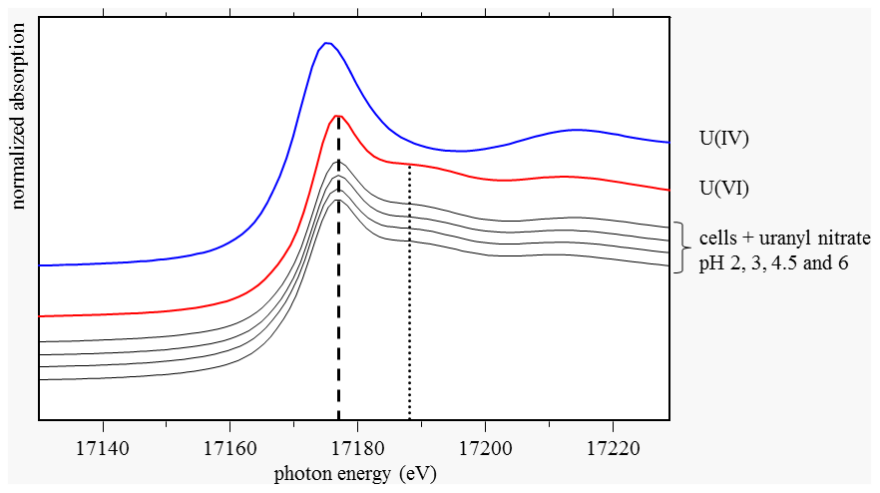
Uranium *L*_III_-edge XANES spectra recorded from the uranium complexes formed by *Paenibacillus* sp. JG-TB8 at pH 2, 3, 4.5 and 6 under anoxic conditions. In addition, the XANES spectra of two solutions, which serve as reference samples for the uranium oxidation states, one of U(VI) and another one of U(IV), each at a concentration of 4×10^−2^ M in 1 M HClO_4_, are shown in the figure. The dashed and the dotted line represent the position of the absorption edge of U(VI) and of the XANES peak resulting from the multiple scattering contribution of the axial oxygen atoms of U(VI), respectively.

In order to obtain information regarding the structure of the formed uranium complexes at molecular scale, XAS measurements were performed. The XANES region of the recorded spectra, located around the absorption edge, was used to check whether a reduction of the added U(VI) to U(IV) occurred. Our results clearly demonstrate that in all samples the bound uranium remained in the oxidation state +6, i.e. it was not reduced (Fig. 2). The presence of U(VI) was clearly confirmed due to the following observations: The absorption edges of all spectra occurred at a photon energy which is typical for U(VI) (Fig. 2, dashed line) and all spectra featured the multiple scattering contribution of the two axial oxygen atoms of U(VI), represented by a XANES peak around 17188 eV (Fig. 2, dotted line). U(VI) reduction was also not observed in parallel samples incubated at a favourable for this strain pH value of 7.2, which supports the finding that the investigated bacterial isolate *Paenibacillus* sp. JG-TB8 was not able to reduce uranium under the studied conditions.

**Figure 3:**
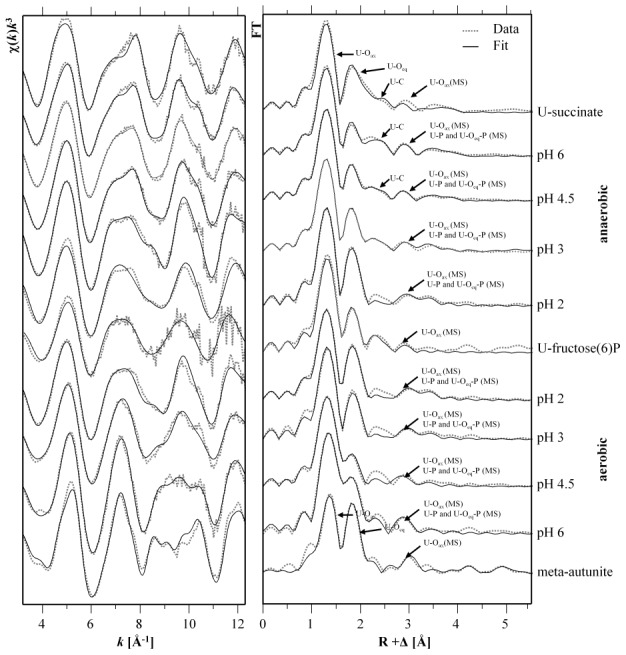
U *L*_III_-edge *k* ^3^-weighted EXAFS spectra (left) and the corresponding Fourier Transforms (right) (3.1 Å^−1^<k<12.4 Å^−1^) of the uranium complexes formed by *Paenibacillus* sp. JG-TB8 at pH 2, pH 3, pH 4.5, and pH 6 at different pH and aeration conditions within 48 hours. For comparison, the spectra of three model compounds, namely uranyl succinate, uranyl-fructose(6)phosphate, meta-autunite are illustrated as well.

**Figure 4:**
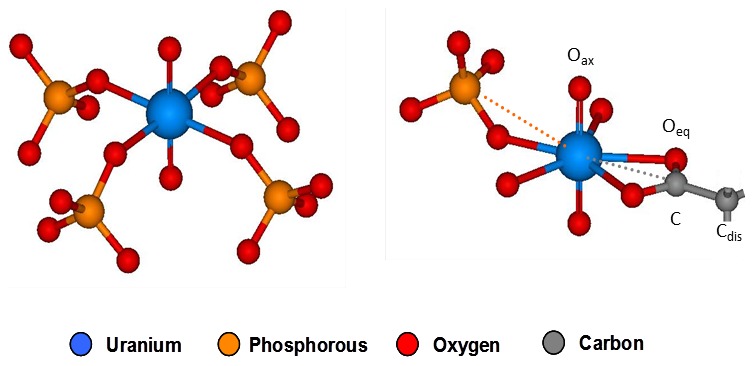
Structural models used for the fitting procedure of the EXAFS spectra obtained from the uranium complexes build by the cells of *Paenibacillus* sp. JG-TB8. The left model created from the crystall structure of meta-autunite was used for the fitting procedure of the sample incubated at pH 6 under oxic conditions. The right model contains fragments of meta-autunite (monodentate coordination at phosphate groups) as well as uranyl triacetate (bidentate coordination to carboxylic groups) and was used for the fitting procedures of all other samples.

The isolated U *L*_III_-edge *k* ^3^-weighted EXAFS spectra and the corresponding FTs of the U(VI) complexes formed by the cells of *Paenibacillus* sp. JG-TB8, along with the best fits are presented in Figure 3. In addition, the spectra of uranyl-fructose(6)phosphate [34], meta-autunite [42] and uranyl succinate [43] as representatives of organic and inorganic uranyl phosphate complexes as well as of organic carboxylate complexes are presented for comparison. The structural data are summarized in Table 4. In agreement with the TRLFS studies, the spectra recorded from the four samples incubated at pH 2 and pH 3 correspond to each other and show a high similarity to that of uranyl-fructose(6)phosphate (Fig. 3). All fits included a shell of two axial oxygen atoms at a radial distance of 1.76 to 1.79 Å, represented by the most prominent FT peak at around R+Δ∼1.3 Å. The second peak of the FTs (R+Δ∼1.8 Å) was related to the backscattering contribution of the equatorial oxygen atoms. The MS path of the axial oxygen atoms, as well as the SS and MS of the phosphate atoms are visible in the FTs in the region between R+Δ = 2.8 and 3.4 Å. The structural parameters of the U(VI) complexes formed in the samples at pH 2 and pH 3 demonstrated an equatorial oxygen shell at a radial distance of 2.35 to 2.36 Å which is typical for a fivefold coordinated uranyl ion [44]. The radial distance of the phosphorous shell at 3.62 Å indicated a monodentate binding of uranyl to phosphate groups. For the sample incubated at pH 3 under anoxic conditions an increased Debye-Waller factor (σ^2^ = 0.0126 Å^2^) was calculated for the equatorial oxygen shell, and it was even higher in the samples incubated at pH 4.5 and pH 6 (σ^2^>0.02 Å^2^), suggesting a structural disorder within this shell. Concomitantly, the radial distance of the U-O_eq_ shell increases with increasing pH values under anoxic conditions from 2.35 Å (pH 2) up to 2.38 Å (pH 6). In accordance to the TRLFS studies, the high Debye-Waller factor revealed a coexistence of uranyl complexes where the uranyl ion was monodentately bound to phosphate groups (averaged U-O_eq_ bond distance = 2.35 Å) and bidentately bound to carboxylic groups (U-O_eq_ bond distance = 2.45 Å [53]). Thus, two shells of carbon atoms (C and distal C, Fig. 4) as well as the strong multiple scattering along the linear U-C-C_dis_ path were included in the shell fitting procedures of the samples incubated at pH 4.5 and 6 under anoxic conditions. In contrast to that, in the oxic sample at pH 6 the radial distance of the equatorial oxygen plane (2.27 Å) was significantly lower compared to the distance calculated for the samples incubated at pH 2 and pH 3. This short radial distance suggested an only fourfold coordinated uranyl ion [44]. In accordance to that, we calculated four equatorial oxygen atoms and, moreover, a comparable number of phosphorous atoms at a radial distance of 3.59 Å, which demonstrated that U(VI) under these conditions was complexed similar to the left model presented in Figure 4. As already suggested by the TRLFS studies, the EXAFS spectrum of the sample incubated at pH 4.5 represents a mixture of the two above discussed complexation types. The high Debye-Waller factor calculated for the equatorial oxygen shell from the complexes formed at pH 4.5 indicates a structural inhomogeneity, caused by a mixture of complexes, exhibiting on the one hand a fivefold coordinated U(VI) bound to organic phosphate groups, and on the other hand U(VI) which was included in a mineral phase complexed by four inorganic phosphate ligands.

**Table 4:** Structural parameters of the uranium complexes formed by the cells of *Paenibacillus* sp. JG-TB8.

Sample	Shell	N^a^	R (Å)^b^	σ^2^ (Å^2^)^c^	ΔE_0_ (eV)
UO_2_-fructose(6)phosphate	U-O_ax_	2.0^d^	1.78(1)	0.001(1)	2.5(8)
pH 3.5 [34]	U-O_eq1_	5.7(9)	2.37(1)	0.016(2)	
pH 2 - oxic	U-O_ax_	2.0^d^	1.77(1)	0.0024(1)	2.4(3)
	U-O_eq_	3.8(2)	2.35(1)	0.0065(4)	
	U-P	2.7(3)	3.62(1)	0.0037(7)	
	U-O-P (MS)	5.4^e^	3.76(1)	0.0037^e^	
pH 2 - anoxic	U-Oax	2.0^d^	1.77(1)	0.0018(1)	2.4(3)
	U-Oeq	4.0(2)	2.35(1)	0.0068(5)	
	U-P	3.1(4)	3.62(1)	0.0044(8)	
	U-O-P (MS)	6.2^e^	3.76(1)	0.0044^e^	
pH 3- oxic	U-O_ax_	2.0^d^	1.78(1)	0.0021(1)	3.4(4)
	U-O_eq_	3.3(2)	2.35(1)	0.0074(7)	
	U-P	1.8(4)	3.62(1)	0.004(1)	
	U-O-P (MS)	3.6^e^	3.76(2)	0.004^e^	
pH 3 - anoxic	U-Oax	2.0^d^	1.76(1)	0.0021(1)	1.6(4)
	U-Oeq	4.7(3)	2.36(1)	0.0126(9)	
	U-P	3.2(3)	3.61(1)	0.0056(9)	
	U-O-P (MS)	6.4^e^	3.75(1)	0.0056^e^	
pH 4.5 - oxic	U-O_ax_	2.0^d^	1.77(1)	0.0025(1)	−1.0(6)
	U-O_eq_	4.2(3)	2.27(1)	0.0103(8)	
	U-P	4.0(3)	3.59(1)	0.008^d^	
	U-O-P (MS)	8.0^e^	3.72(1)	0.008^e^	
pH 6 - oxic	U-O_ax_	2.0^d^	1.79(1)	0.0016(1)	2.7(4)
	U-O_eq_	3.9(2)	2.27(1)	0.0037(3)	
	U-P	4.1(3)	3.59(1)	0.008^d^	
	U-O-P (MS)	8.2^e^	3.72(2)	0.008^e^	
meta-autunite [42]	U-O_ax_	2.2(1)	1.76	0.0045	−11.0
	U-O_eq_	3.9(2)	2.29	0.0026	
	U-P	2.3(3)	3.60	0.008^d^	
pH 4.5 - anoxic	U-Oax	2.0^d^	1.77(1)	0.0021(1)	0.7(3)
	U-Oeq	6.6(5)	2.36(1)	0.020(1)	
	U-P	1.8(2)	3.60(1)	0.004(1)	
	U-O-P (MS)	3.6^f^	3.72(1)	0.004^e^	
	U-C	1.9(1)	2.88(1)	0.0042^d^	
	U-C_dis_	1.9^f^	4.35(1)	0.00645^d^	
pH 6 - anoxic	U-Oax	2.0^d^	1.77(1)	0.0023(1)	0.4(4)
	U-Oeq	5.7(9)	2.38(1)	0.022(1)	
	U-P	2.1(2)	3.60(1)	0.0034(1)	
	U-O-P (MS)	4.2^f^	3.72(1)	0.0034^e^	
	U-C	2.7(2)	2.89(1)	0.0042^d^	
	U-C_dis_	2.7^f^	4.35(1)	0.00645^d^	
Uranyl succinate	U-Oax	2.0^d^	1.776(2)	0.0014(1)	4.5(5)
pH 4.46 [43]	U-Oeq	5.0(4)	2.449(5)	0.0089(9)	
	U-C	2.6(3)	2.888(8)	0.0042^d^	
	U-C_dis_	2.6^f^	4.35(1)	0.00645^d^	

Standard deviations as estimated by EXAFSPAK are given in parenthesis.

a Errors in coordination numbers are ±25%.

b Errors in distance are ±0.02 Å.

c Debye-Waller factor.

d Parameter fixed for calculation, Debye-Waller factor of the U-P, U-C and U-Cdis path were fixed acccording to the Debye-Waller factors calculated for the corresponding model compounds (see references 42 and 43).

e Coordination number linked twice and Debye-Waller factor once to the N and σ^2^ of the U-P path.

f Coordination number (N) linked to N of U-C_1_ path.

### TEM analyses

**Figure 5:**
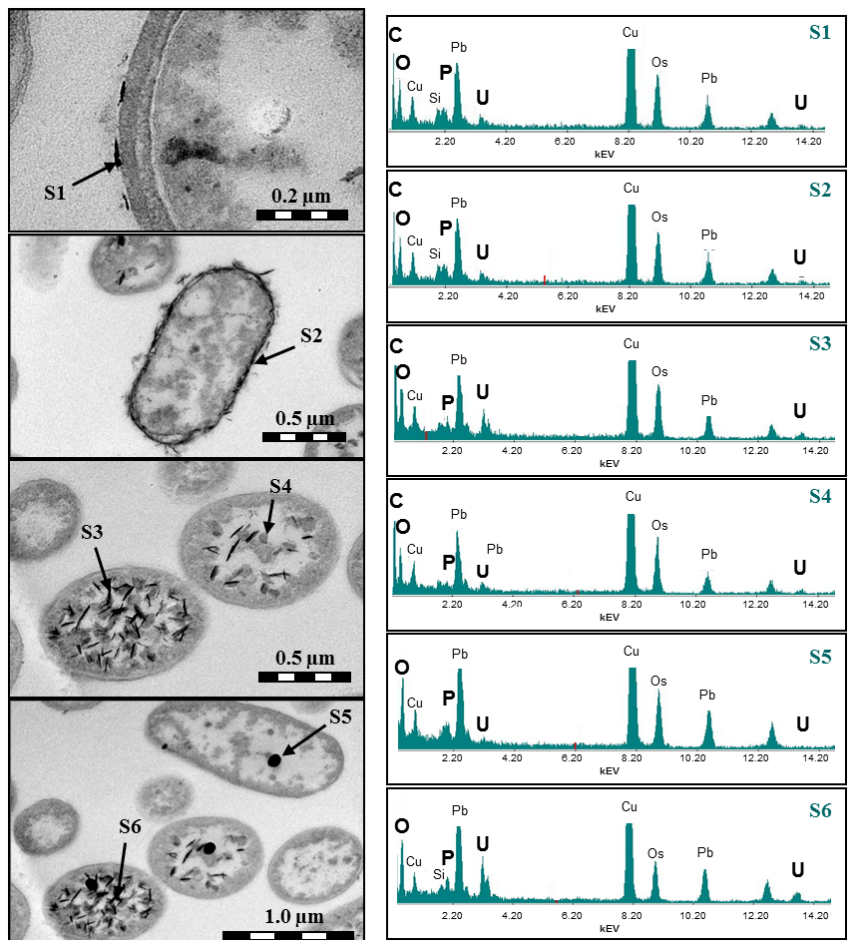
Transmission electron micrograph (left) of uranium precipitates deposited by the cells of *Paenibacillus* sp. JG-TB8 at pH 4.5 under oxic conditions. Energy-dispersive X-ray spectra (right) of the sample points (S1–S6) are marked with arrowheads.

The uranium associated with the cells was localized using transmission electron microscopy combined with EDX. No visible uranium precipitates were detected in the anoxic samples as well as in the oxic samples incubated at pH 2, pH 3 and pH 6. However, in the sample incubated at pH 4.5 under oxic conditions many electron-dense uranium precipitates recognizable as high-contrasting spots were detected. Uranium was located at the cell surface as well as intracellularly in a form of needle-like fibrils, associated with cell compounds or polyphosphate granules (Fig. 5). An intracellular uranium accumulation in polyphosphatic granules has also been demonstrated for *Pseudomonas aeruginosa* [45]. The lower uranium concentration in the sample incubated at pH 6 was the most likely reason for the failed detection of uranium precipitates at this pH.

EDX analyses demonstrated that the electron dense precipitates contained different amounts of uranium (U). Besides that, typical energy-lines for phosphorous (P) and oxygen (O) were observed, indicating formation of uranyl phosphate complexes (Fig. 5) and confirming therewith the above presented spectroscopic results. The peaks for C, Cu, Os, Pb, and Cl were a result of the sample preparation and the copper grid used to support the ultra-thin cell sections. The presence of silicon results from the oil in the diffusion pump of the column of the used TEM system.

### Live/Dead staining

**Figure 6:**
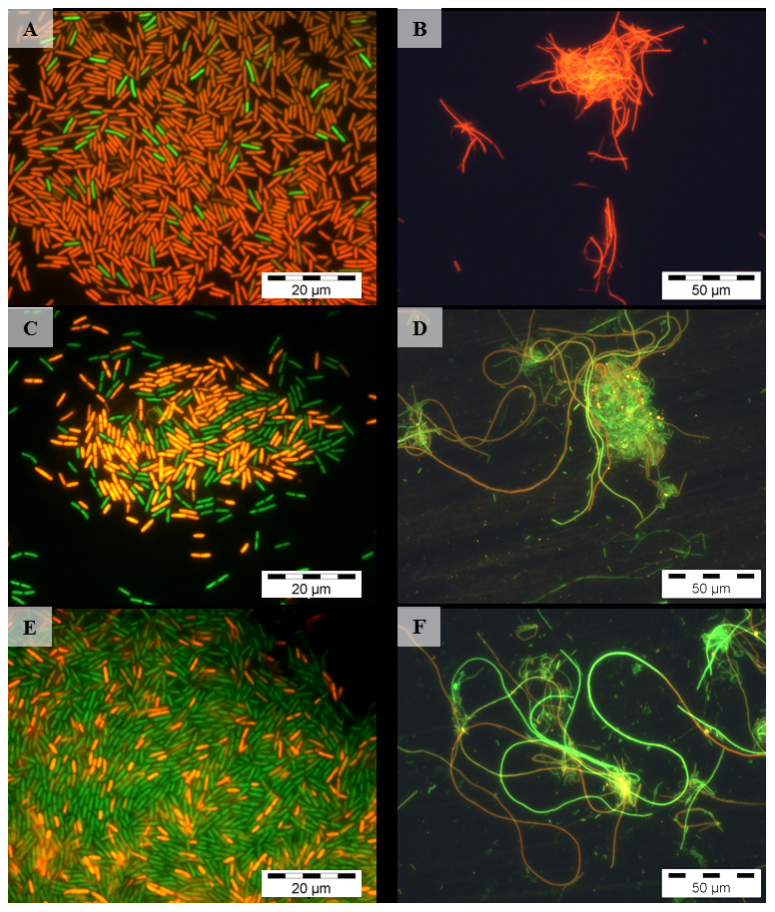
Representative microscopic pictures of *Paenibacillus* sp. JG-TB8, stained with the Live/Dead Kit after the treatment with uranium under aerobic (left) and anaerobic (right) conditions at pH 3 (A, B), pH 4.5 (C, D), and pH 6 (E, F) for 48 hours. Pictures were taken in fluorescence mode using a fluorescence mirror unit (U-MSWB; Olympus Europa Holding GmbH, Hamburg, Germany) with excitation wavelengths between 420 and 460 nm.

For all studied pH values and aeration conditions, parallel samples with and without uranium exhibit no significant differences, indicating that uranium was not responsible for the cell damage, but cell viability was mainly affected by the pH conditions. Under oxic conditions at pH 6, 83% of the cells were alive, whereas the fraction of the living cells decreased with decreasing pH due to the increasing distance from the preferred pH conditions of the strain (Fig. 6). Hence, at pH 4.5 and pH 3 only 43% and 5% of the cells were viable, respectively. At pH 2 all cells showed damaged cell membranes and stained red. Investigation of the cell viability in the anoxic samples showed that the proportion of living cells in samples incubated at pH 4.5 (82% living cells) and pH 6 (87% living cells) did not much differ from each other. In contrast to that, at pH 3 and pH 2 (anoxic conditions) all cells stained red, indicating damaged cell membranes and, thus, dead cells.

### Phosphate metabolism

The measured phosphatase activity of *Paenibacillus* sp. JG-TB8 in dependency on the pH value and the aeration conditions is summarized in Table 5. Under oxic as well as under anoxic conditions the phosphatase activity increased with increasing pH. The highest activity was calculated under both aeration conditions for the sample incubated at pH 6, which was not surprising as this pH was the closest one to the pH optimum of the strain. However, it is evident from the data that the phosphatase activity of the strain, in particular under moderate acidic conditions (pH 4.5 and pH 6) was significantly reduced under anoxic conditions. At pH 6 the phosphatase activity of the aerobically grown cells was more than 20 times higher than under anoxic conditions.

**Table 5:** Phosphatase activity of *Paenibacillus* sp. JG-TB8 incubated at different pH values and aeration conditions.

pH	Phosphatase activity [Units/g cells]	
	Oxic	Anoxic
2.0	0.009±0.003	0.007±0.002
3.0	0.026±0.005	0.014±0.003
4.5	0.239±0.016	0.017±0.003
6.0	0.62±0.095	0.028±0.003
Dead cells (pH 6)	0.004±0.001	

**Figure 7:**
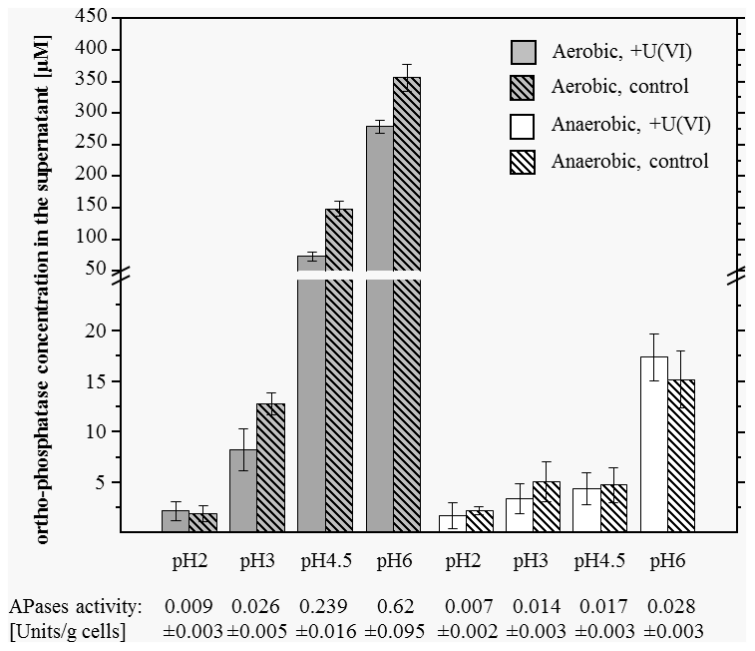
Amount of orthophosphate in the supernatant of cells of *Paenibacillus* sp. JG-TB8 after an incubation of 48 hours in dependency on pH, the aeration conditions and the presence of uranium.

As shown in Figure 7 the reduction of the enzymatic activity under anoxic conditions dramatically influenced the release of orthophosphate. Under anoxic conditions only very low amounts of orthophosphate (<16 µM) were determined in the samples. In accordance to the phosphatase activity studies, a comparable low amount of orthophosphate was measured in the samples incubated at pH 2 and pH 3 under oxic conditions. In contrast to that, the orthophosphate concentration in the oxic samples, exhibiting a high phosphatase activity, was about 150 µM (pH 4.5) and 350 µM (pH 6), respectively. No differences in the orthophosphate content were observed between the uranium-treated and untreated cell samples under anoxic conditions. This was also the case for the cells treated under oxic conditions at pH 2 and pH 3 (Fig. 7). In contrast to that, significant differences were observed in the orthophosphate concentration between the uranium-treated and untreated cell samples under oxic conditions at pH 4.5 and pH 6. This removal of orthophosphate from the supernatant was caused by the precipitation of inorganic uranyl phosphate complexes at both pH values.

## Discussion

The bacterial isolate *Paenibacillus* sp. JG-TB8 used in this study was isolated from an anaerobic consortium, enriched from a soil sample with moderate acidic pH of about 4.5 from the uranium mining waste pile “Haberland” located in Saxony, Germany [25]. The strain JG-TB8 possessed the ability to switch between aerobic and anaerobic growth. When cultivated aerobically JG-TB8 formed endospores and the cells had a length of 3 to 6 µm. In contrast to that under anaerobic growth conditions the cells of JG-TB8 exhibited a filamentous phenotype. A comparable filamentous phenotype has been described for *ftsH*-null-mutants of *Bacillus subtilis* [46]. Interestingly, the *ftsH* knockout mutants were also unable to sporulate. Further studies on the anaerobic growth of *B. subtilis* indicated that the same protein, *ftsH*, is essentially required for the fermentation process [47] implicating a strong connection between the anaerobic metabolism and spore formation as well as the occurrence of a filamentous phenotype.

### Interactions with U(VI) under highly acidic conditions (pH 2 and pH 3)

According to calculations with the software package “EQ3/6” [48] using equilibrium constants and uranyl hydrolysis constants of the NEA database published by Guillaumont and co-workers [49], U(VI) existed in the uranium solutions at pH 2 and pH 3 almost exclusively (>98%) in a form of uranyl ion which is soluble, bioavailable and highly toxic.

Among all four samples (pH 2 and pH 3, oxic and anoxic) we observed a rapid association of U(VI) with the bacterial cells, which is a characteristic feature of biosorption processes [8]. Comparing the binding capacities of the studied bacterial cells after one hour of incubation under the two different aeration conditions, it is evident that more uranium was associated with the cells under oxic than under anoxic conditions (Table 1). The spectroscopic results consistently demonstrate that at pH 2 and pH 3 the complexation of uranium exclusively occured *via* phosphate groups of organic origin under both aeration conditions. The important role of organic phosphate groups as ligands for the U(VI) complexation at pH 2 and pH 3 has already been demonstrated for other bacterial [50]–[51] as well as archaeal strains [27]. Although uranium was bound by JG-TB8 at pH 2 and pH 3 (Table 1), we were not able to detect uranium microscopically, neither in the samples treated at oxic, nor in the samples treated under anoxic conditions. The most likely reason for this was the detection limit of EDX. Whereas bigger uranium precipitates could be detected easily, due to their high amount of uranium, it was not possible to detect smaller precipitates or equally-distributed uranium complexes formed at the negatively charged functional groups of the cell surface.

### Interactions with U(VI) under moderate acidic conditions (pH 4.5 and pH 6)

Under moderate acidic conditions (pH 4.5 and pH 6), which are characteristic for many uranium-contaminated sites, the speciation of U(VI) differed from that at pH 2 and pH 3. According to calculations with the software “EQ3/6”, at pH 4.5 only about 57% of the U(VI) was present in form of the uranyl ion (UO_2_^2+^) and the formation of uranyl hydroxide complexes in the solution was favored - 26% (UO_2_)_2_(OH)_2_^2+^, 6% (UO_2_)_3_(OH)_5_^+^. At pH 6 uranium hydroxide - 61% (UO_2_)_3_(OH)_5_^+^, 12% (UO_2_)_4_(OH)_7_^+^ - and uranyl carbonate complexes - 15% (UO_2_)_2_CO_3_(OH)_3_^−^ - dominated the uranium speciation.

The amounts of U(VI) accumulated by the cells at pH 4.5 after one hour were higher compared to those observed at pH 2 and pH 3. This was caused by complexation of U(VI) at additional deprotonated and therefore binding-capable functional groups at the cell surface. In particular, as demonstrated by Fowle and colleagues the carboxylic groups of the cell surface are involved in the uranium complexation at moderate acidic pH values [52]. The efficient uranium binding by microorganisms is in general attributed to the large number of uranium-binding ligands on their thick (Gram-positive bacteria) or rather complex and multilayer (Gram-negative bacteria) cell wall structures [53]. The cell wall of the studied here Gram-positive *Paenibacillus* sp. JG-TB8 strain is rich in carboxylic and phosphate groups provided by the peptidoglycan layer and the teichoic acids.

We furthermore demonstrated that under moderate acidic conditions the availability of oxygen strongly influenced the cell metabolism of JG-TB8, which in turn influenced the speciation of uranium associated with the bacterial cells. Under oxic conditions enhanced formation of inorganic uranyl phosphate mineral phases was observed. TRLFS and XAS analyses demonstrated that at pH 4.5 the major part, and at pH 6 the total amount of the added uranium was precipitated within 48 hours in meta-autunite-like mineral phases. In the samples treated with uranium at pH 4.5 under oxic conditions the spectroscopic data revealed a mixture of organic and inorganic uranyl phosphate complexes. However, the low radial distance of the U-O_eq_ shell (R = 2.27 Å) indicated that most of the uranium present in this sample existed in the uranyl phosphate mineral form. This finding is in contrast to the TRLFS results, where the luminescence emission maxima correspond better to those of organic uranyl complexes. However, the overestimated amount of the latter by TRLFS data was caused by the unequal luminescence properties of organic and inorganic uranyl phosphate complexes. We calculated by using the luminescence data and the amount of accumulated U(VI) of the oxic samples incubated at pH 2 (exclusively organic uranyl phosphate complexes) and pH 6 (exclusively inorganic uranyl phosphate complexes) that the luminescence emission of the uranyl mineral phases was more than ten times lower, compared to those of the organic uranyl phosphate complexes (Figure S3 and Table S1 in File S1). TEM studies of the sample incubated at pH 4.5 demonstrated that uranium was located at the cell surface as well as intracellularly. Such intracellular uranium deposits were detected only in about 30% of the cells and exclusively in those cells which exhibit damaged cell membranes, which suggested that the formation of intracellular uranium deposits was a consequence of increased cell permeability after cell death. This observation supported the finding of Boswell and colleagues [54] who demonstrated that U(VI) cannot enter intact cells.

TRLF spectroscopic analyses of the bacterial cell samples, grown anaerobically and treated with U(VI) under anoxic conditions demonstrates the formation of uranyl carboxylate complexes under these conditions (Fig. 1, Table 2). These complexes are known to show weak/no luminescence at room temperature. However, recent studies demonstrated that U(VI) complexes formed at carboxylic groups of peptidoglycan, the major cell wall compound of the studied bacterium, are luminescent at room temperature [39]. The calculated lifetimes of the uranyl carboxylate complexes (τ_5_ = 7.8±1.3 µs and 9.2±1.1 µs in the sample incubated at pH 4.5, and pH 6, respectively) were in line with that of the 1∶1 uranyl carboxylate complex studied recently [39]. Moreover, comparable results which suggested the formation of uranyl carboxylate complexes were obtained by spectroscopic studies of the U(VI) complexes formed at the cells of the acidothermophilic crenarchaeon *Sulfolobus acidocaldarius* [27] (Table 2). The exclusive binding of U(VI) at organic functional groups under anoxic and moderate acidic pH conditions is in contrast to all studies, which were performed on U(VI)/microbe interactions under anoxic conditions. So far either a U(VI) reduction to U(IV) [55]–[57] or a precipitation in uranyl phosphate minerals under anoxic conditions [18], [58] has been described.

### Key role of phosphatase activity for uranium complexation

The observed formation of uranyl phosphate mineral phases by microbial cells has also been described in several previous studies [14], [17], [19], [49], [59]. In most cases the release of orthophosphate, which is involved in the uranium precipitation, was attributed to the activity of various phosphatases, which release inorganic orthophosphate from organic phosphate compounds. Corresponding enzyme activities had been described for a large variety of aerobic and anaerobic bacteria as well as in archaea [20]–[22], [60]. It has been demonstrated that in the presence of an organic phosphate source a bacterial phosphatase, which was over expressed in *Pseudomonas* strains, could release sufficient amounts of orthophosphate to precipitate uranium even from low concentrated (2·10^−5^ M uranium) solutions [61]. Moreover, recent studies postulated, that U(VI) biomineralization based on the hydrolization of organophosphate by heterotrophic bacteria is a promising bioremediation strategy to immobilize uranium [17] and that even under anoxic conditions the U(VI) phosphate biomineralization has a higher immobilization potential than the bioreduction to U(IV) [62]. We observed that under moderate acidic conditions the phosphatase activity of *Paenibacillus* sp. JG-TB8 and the corresponding release of orthophosphate were strongly reduced under anoxic conditions. This finding is in contrast to the results of Beazley and colleagues, who found an only slightly reduced orthophosphate release by the facultative anaerobic bacterium *Rahnella* sp. Y9602 under anaerobic conditions after incubation for 12 hours, resulting in quantitatively similar uranyl phosphate precipitation [18]. Moreover, the observed U(VI) precipitation rate by this *Rahnella* species during the first hour of incubation was even higher under anaerobic conditions. The observed reduction of the phosphatase activity of JG-TB8 is also in contrast to studies using isolated enzymes (acid and alkaline phosphatases), where the specific enzyme activity did not change significantly [63], and the phosphatase activity of *E. coli*, which was shown to be dramatically enhanced after a shifting from oxic to anoxic conditions [20]. The reasons for the reduction of the phosphatase activity of Paenibacillus sp. JG-TB8 under anaerobic conditions may include the suboptimal pH for the produced phosphatase(s), or a lack of active co-factors (metals, other organic co-factors) necessary for enzyme activity, under these conditions. Missing organophosphates as substrate for the phosphatase(s), however, could be excluded in this respect as for the activity studies *p*-nitrophenyl phosphate was provided as phosphate source.

The orthophosphate release in the samples corresponds well to the phosphatase activity (Fig. 7). However, for the release of orthophosphate by microbial phosphatases organic phosphate sources are needed as substrate. As we did not provide any additional phosphate source, the only possible origin of phosphate-containing organic substrates were the released cell compounds of the damaged cells. Live/Dead staining demonstrated that about 10% and 57% of the cells in the oxic samples incubated at pH 6 and pH 4.5, respectively, showed damaged cell membranes (Fig. 6). The comparison of the control versus the uranium-treated cell samples along all pH- and aeration conditions also demonstrated that a significant reduction of the orthophosphate due to the addition of uranium was only observed in the oxic samples incubated at pH 4.5 and pH 6. This reduction additionally confirmed the uranium biomineralization by the orthophosphate released due to the phosphatase activity. Nevertheless, the observed rapid association of uranium with the cells at both pH values (Table 1) suggested an initial U(VI) binding to functional groups at the cell surface, which subsequently served as nucleation sites for metal precipitation and biomineralization. A corresponding mechanism has also been suggested for the radionuclide accumulation by *Rahnella* sp. [18] and *Citrobacter* sp. [64].

## Conclusions

The present study demonstrates that U(VI) was complexed by the cells of the Gram-positive bacterium *Paenibacillus* sp. JG-TB8 under highly acidic conditions (pH 2 and pH 3) exclusively *via* organic phosphate groups independently on the aeration conditions applied for growth and treatment with uranium. In contrast to that under moderate acidic conditions, which are relevant for many uranium contaminated sites, the immobilization of uranium depended on the pH (pH 4.5 or pH 6) and also on the aeration conditions. JG-TB8 precipitated significant amounts of U(VI) in uranyl phosphate mineral phases under moderate acidic and oxic conditions, whereas under anoxic conditions no corresponding precipitation occurred due to the dramatic inhibition of the phosphatase activity of the cells. As we added, in contrast to other studies, no additional phosphate source, the organic phosphates needed for the enzymatic release of orthophosphate were provided by the part of the cells which degraded during the incubation with uranium. Hence, this study demonstrates that the metabolism of the facultative anaerobic strain *Paenibacillus* sp. JG-TB8, which is indigenous in uranium-contaminated sites, underwent aeration-dependent changes, which strongly influenced the capability to immobilize U(VI). The immobilization ability of the strain was connected to the phosphatase activity of the cells, which in turn was strongly influenced by the aeration conditions. The results of this study contribute to a better understanding of the uranium mobility in contaminated ecosystems and suggest an air ventilation of uranium-contaminated sites as a possible eco-management option to improve the conditions for U(VI) biomineralization.

## Supporting Information

File S1 Contains Figure S1, Phylogenetic classification of *Paenibacillus* JG-TB 8. Figure S2, Light microscopic pictures of *Paenibacillus* sp. JG-TB8. Figure S3, U(VI) luminescence spectra of organic and inorganic uranyl phosphate complexes. Table S1, U(VI) luminescence intensities of organic and inorganic uranyl phosphate complexes.
(DOCX)
