# Supplementary material for: Decrease of U(VI) Immobilization Capability of the Facultative Anaerobic Strain Paenibacillus sp. JG-TB8 under Anoxic Conditions Due to Strongly Reduced Phosphatase Activity
Source: PLoS One. 2014 Aug 26;9(8):e102447. doi: 10.1371/journal.pone.0102447 (PMC4144796; doi:10.1371/journal.pone.0102447)
Supplement: File S1 — Contains Figure S1, Phylogenetic classification of Paenibacillus JG-TB 8. Figure S2, Light microscopic pictures of Paenibacillus sp. JG-TB8. Figure S3, U(VI) luminescence spectra of organic and inorganic uranyl phosphate complexes. Table S1, U(VI) luminescence intensities of organic and inorganic uranyl phosphate complexes. (DOCX) [file pone.0102447.s001.docx]

**Supporting Information**

**Figure S1.** Phylogenetic dendrogram of 16S rRNA gene sequence retrieved from *Paenibacillus* JG-TB 8

The dendrogram was constructed using neighbourjoining method, based on sequence comparison of the region corresponding to the *E. coli* 16S rRNA gene positions 101 to 1477) and rooted with the 16S rRNA gene sequence of the typus strain *Paenibacillus borealis* DSM 13188^T^. The scale bar represents a 1% difference in nucleotide sequences.

**
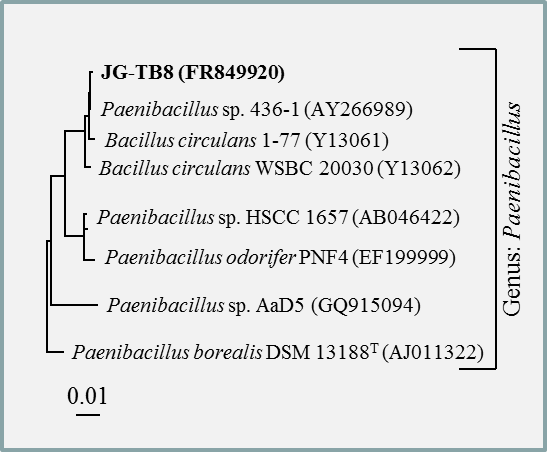
**

**Figure S2.** Light microscopic pictures of *Paenibacillus* sp. JG-TB8 grown aerobically in liquid NB medium (A1,A2), and anaerobically in ATCC medium 591 (B1,B2).


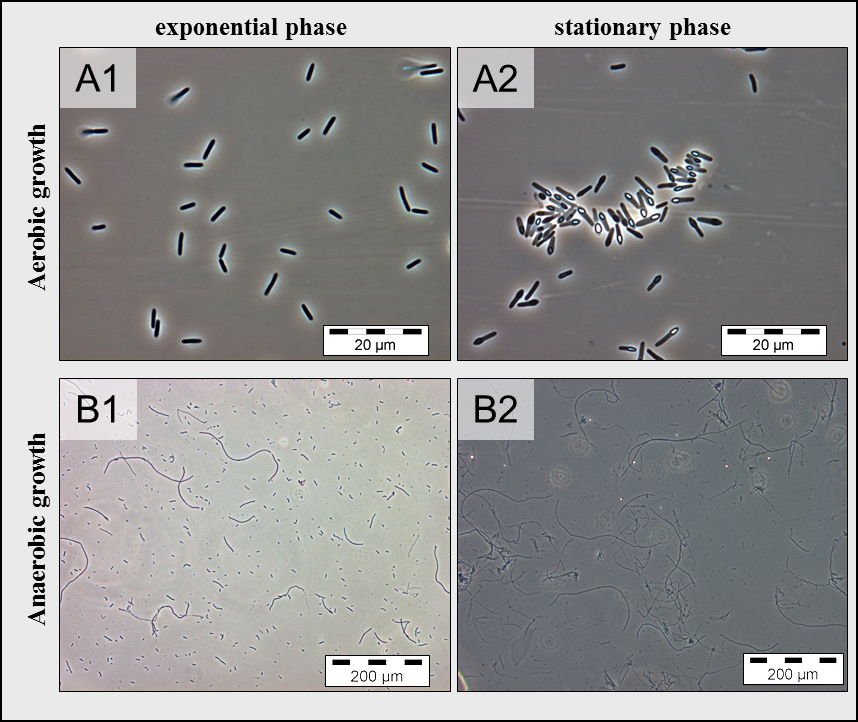


**Figure S3.** U(VI) luminescence spectra (not normalized) recorded from the organic uranyl phosphate complexes formed at pH 2 and the inorganic uranyl phosphate complexes formed at pH 6under oxic conditions within 48 hours by the cells of *Paenibacillus* sp. JG-TB8.


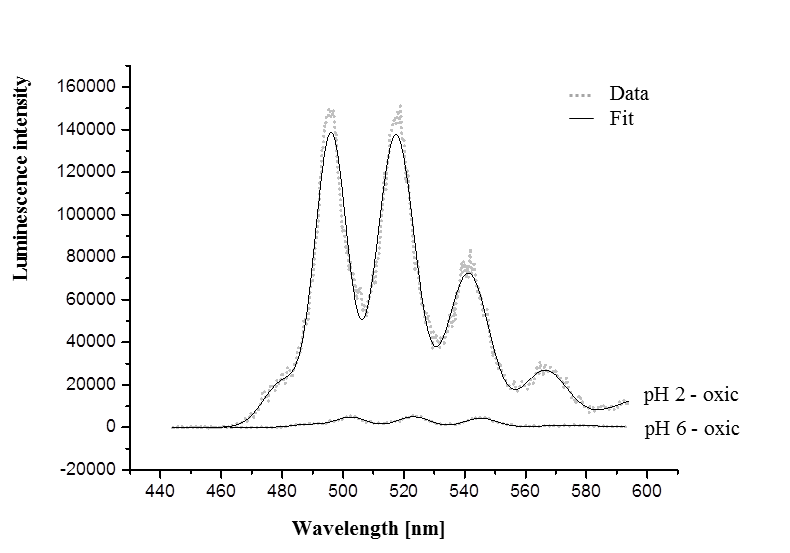


**Table S1.** U(VI) luminescence properties of the organic uranyl phosphate complexes formed at pH 2 and the inorganic uranyl phosphate complexes formed at pH 6under oxic conditions within 48 hours by the cells of *Paenibacillus* sp. JG-TB8.

| **Experimental conditions** | **Luminescence intensity maximum** | **U(VI) binding capacity [mg/g_dry biomass_]** | **Ratio**  **Luminescence:U(VI)** |
| --- | --- | --- | --- |
| JG-TB8 – pH 2.0 – oxic conditions - organic uranyl phosphate complexes | 151000 | 63 | 2397 |
| JG-TB8 – pH 6.0 – oxic conditions – inorganic uranyl phosphate complexes | 5500 | 24 | 229 |
